# A Conceptual Review of Teacher Enthusiasm and Students' Success and Engagement in Chinese EFL Classes

**DOI:** 10.3389/fpsyg.2021.742970

**Published:** 2021-09-03

**Authors:** Chunju Peng

**Affiliations:** ^1^English Department, Hainan Medical University, Haikou, China; ^2^Department of Postgraduate Studies, Faculty of Education, Languages and Psychology, SEGi University, Kota Damansara, Malaysia

**Keywords:** Chinese EFL classes, students' success, engagement, teacher enthusiasm, learner-eccentric learning

## Abstract

The swift growth and progress of colleges and universities across China noticed a request for teaching and learning English as Foreign Language (EFL), and regarding the quality of higher education, student engagement has been at the center of attention which has a remarkable role due to the arrival of positive psychology in language learning recently. To this end, on the one hand, nurturing student engagement in EFL classes corresponds to requests from the recent national university English curriculum selected in 2015 in China. On the other hand, a bulk of studies has acknowledged difficulties that hinder the construction of a learner-centric learning situation. Moreover, there is a dearth of inquires which have focused on teachers' role in general and affective aspects namely enthusiasm, in particular. According to the literature review, the definition of these constructs, namely teacher enthusiasm and students' success and engagement are presented. In a nutshell, the implications for teachers, university administrators, teacher-trainers, and future researchers are presented, and new directions for future research are allocated.

## Introduction

Since the mid-1990's, the rapid development of the higher education system in China has caused a chain of difficulties, such as a decrease in educational expenses for each student, failing teaching circumstances, and extensive variants in teaching quality among the institutions (Wang, 2017). Accordingly, there has been an obvious failure in general quality, and many teachers and scholars have recently articulated apprehensions about the quality of teachings; therefore, higher education in China is shifted from quantity extension to quality improvement (Yin and Wang, 2016).

Although the teaching quality was monitored by the Chinese Ministry of Education, the reported issues are related to some significant determinants manipulating teaching, such as accommodations, tools, and the guidelines of teaching, in preference to the factors associated with the reality of teaching such as teachers' teaching tactics, methods, and the characteristics of stakeholders such as students and teachers (Yin and Wang, 2016). For instance, great attention has been paid to emotion in previous years due to the dominance of positive psychology in language learning, and researchers approved that learners encounter different types of emotions in language learning (Dewaele and Li, 2020), so emotions have an indispensable role in language learning process and success (Li, 2020). The emotional scale has shifted its priority from the traditional concern in anxiety to embrace elements of positive psychology such as pleasure, enthusiasm, hope, happiness, appreciation, etc. (MacIntyre et al., 2019).

Moreover, one of the predominantly significant positive psychosomatic constructs in education is engagement for its relations to different student results containing success, fulfillment, and motivation (Neel and Fuligni, 2013). Oga-Baldwin (2019) pinpointed that the research in the student engagement domain focused on two aspects, namely affective factors, and social factors. While the former is related to factors such as motivation, emotion, attentiveness, the latter refers to the antecedents of student engagement and environmental factors such as teacher and peer relations. The influences of individual and environmental determinants on student engagement are addressed by Mercer (2019) as she declared that those students who felt knowledgeable, independent, and have positive interactions with their peers and teachers, enjoy learning more, and then turn into a more interested and engaged person in the language course (Hiver et al., 2021).

Recently, while it is indicated that many issues are determining the learning procedure and the quality of learners, more attention is being devoted to the conception of how social environments are associated with student engagement (Shoshani and Eldor, 2016). The classroom is the cornerstone for accomplishing a learner-centric, involving learning setting that is representative of increasing their attentiveness (Exeter et al., 2010). Student engagement is reflected as a flexible process powerfully influenced by the parents, school, and society, which sequentially support students' cognition, emotion, and performance (Lam et al., 2012).

Among those contextual elements such as teachers, peers, parents, and society, the teacher takes on a fundamental role in affecting student engagement and success and can enlighten many of the discrepancies frequently detected within stages of classroom engagement (Pishghadam et al., 2021). Similarly, teachers' use of cooperative teaching styles along with interpersonal interaction motivates student engagement (Umbach and Wawrzynski, 2005; Xie and Derakhshan, 2021). Indeed, the success of the teaching is influenced by the teachers' qualifications in controlling and implementing the system (Çevik Kiliç, 2018); however, to date, the role of teachers' positive emotions on student engagement has not been scrutinized.

One of these constructive positive emotions is teacher enthusiasm that has been regarded as a central characteristic of an effective teacher and also a predictor of student learning behavior, emotional states, and presentation (Lazarides et al., 2019). Teacher enthusiasm is interpreted as a conjoined manifestation of positive emotional involvements and the behavioral display of these involvements in teaching (Keller et al., 2018) that has constructive effects on students' motivational, emotional, and social consequences, other than their academic success.

Similarly, teacher enthusiasm is being defined as nonverbal communication that the teacher replicates (Baloch and Akram, 2018); teacher competence and effective teacher features are based on positive emotions (Keller et al., 2018), and positive emotional involvements happen while acting out their responsibilities (Keller et al., 2014). The teacher's enthusiasm positively affects learning success (Cui et al., 2017). The positive consequences of teacher enthusiasm are also in proportion to the standard valuable effects of positive emotions in improving presentation, success, and fulfillment (Keller et al., 2014). Even though researchers have not investigated a relationship between teacher enthusiasm and student engagement, previous research indicated that motivated teachers activate student attention, stimulate learners to be more interested and engaged, and encourage them to be attentive (Zhang, 2013).

Most of the studies carried out on student engagement originated from research in the United States (Shaw et al., 2015; Witkowski and Cornell, 2015). Although student engagement is valued among Chinese teachers (Zhu and Arnold, 2013; Wang, 2017) thanks to its high relationship with teaching success and improvement (Yin and Wang, 2016), just a few studies have been done in China to explore student commitment. For instance, Zhang et al. (2015) investigated student engagement in China from the students' perceptions. Accordingly, there is a request for more research on the role of English teacher enthusiasm in involving students in classrooms in a Chinese setting.

## Teacher Enthusiasm

Since enthusiasm is hypothesized as an emotional construct, derived from the positive emotion in general and the intrinsic motivation domain in particular (Kunter and Holzberger, 2014). Enthusiasm can boost a wide range of teaching and learning consequences, as well as teaching success, students' presentation, and motivation (Hsu, 2010). This construct was eventually defined as enthusiastic teaching behaviors, which were reflected as a central predictor of teaching superiority and efficacy (Patrick et al., 2000). Since teacher enthusiasm supports the teacher's active interaction with students, it is called teacher immediacy, as an indefinable construct that has been regarded as nonverbal behaviors that allude to physical and emotional intimacy between individuals (Keller, 2011). Enthusiastic teachers use humor to make learning enjoyable and entertaining (Frenzel et al., 2009) that it can be followed by teachers talk with a smile on their face that cause confidence in learners that makes the classroom setting sociable and collaborative that is related to the nonverbal immediacy (Derakhshan, 2021).

Researchers describe teacher nonverbal immediacy as a variety of behaviors that increase psychological closeness between communicators including eye contact, facial expressions, tone of voice, posture, and movement (Pogue and AhYun, 2006). An interested teacher often vitalized the class with pleasure, satisfaction, and eagerness involves learners to take part and inspires them to explore (Patrick et al., 2000).

## Implications and Future Directions

As has been mentioned in the literature review, teacher enthusiasm is considered as a vital teacher distinctive feature that reveals its significant role on student inspiration and engagement through sympathetic instructional practices such as support for independence, aptitude, social empathy, and subject relevance. Engagement engenders learning and envisages learning success, and it is regarded as one of the solutions for the problems of students' disengagement such as low accomplishment and great dropout rates. In the class of an enthusiastic teacher, learners feel more autonomous and less concerned (Cui et al., 2017). When students feel confident in the class, they are ready to take educational and emotional risks, so they become self-confident enough to be engaged in the route of learning (Ulmanen et al., 2016). Indeed, building a warm learning milieu without any criticism sets grounds to nurture student engagement. So it is recommended for teachers who are caring about the quality of their classroom to increase students' sense of belonging through interaction and immediacy that lead to students' emotional engagement. Moreover, the teacher enthusiasm can boost students' attentiveness and their inclination and willingness to learn that can be employed through tasks that trigger extrinsic rewards, fulfill learners' intrinsic well-being, and provide a sense of possession and autonomy to students that all lead to a cognitive engagement (Skinner and Pitzer, 2012).

Regarding the manifestation of teacher, it is worth mentioning that enthusiasm might be also significant for the teachers themselves which bring about work-related well-being of a teacher as enthusiastic teacher is inclined to be more fulfilled in their life and at the work (Burić and Moe, 2020).

Enthusiasm is two sides of the coin; one side is behavioral aspects that indicate nonverbal forms of teacher immediacy, such as tone, facial motions, comicality, eye contact, smiling, and body movement, a sympathetic and caring classroom setting (Witt and Wheeless, 2001) where students feel motivated (Derakhshan, 2021). Nonverbal immediacy behaviors in interactions are rewarding that inspire students to be more attentive during class instruction (York, 2013). Teachers' immediacy behaviors can contribute to teaching efficiency through preserving learner attention and inhibiting boredom and consequently it may raise student motivation as a purpose of teacher's enthusiasm (Babab, 2007; Wang and Derakhshan, 2021). The other side of enthusiasm is behavioral in which the teacher may transfer enthusiasm by verbal signals and prompts, such as mentioning the value of the learning subject matter or articulating teachers' interest (Patrick et al., 2000).

Given the prominence of teacher enthusiasm for both students and teachers, teacher trainers and Chinese university managers should concentrate on promoting interest and pleasure in teaching and the subject matter. Furthermore, a situation without stressful working situations is required for the teachers that allow sustaining enthusiasm and by cultivating teacher enthusiasm, a viable positive result on teaching quality, teacher well-being, student motivation, engagement, and learning can be accomplished.

In summary, future studies on students' engagement are required to explore the most engaging approaches and motivational directions regarding teachers' enthusiasm, differentiating between enthusiasm for the subject and teaching. So, future researchers should run studies that consider both dimensions of teacher enthusiasm to add new perceptions in this field.

## Author Contributions

The author confirms being the sole contributor of this work and has approved it for publication.

## Conflict of Interest

The author declares that the research was conducted in the absence of any commercial or financial relationships that could be construed as a potential conflict of interest.

## Publisher's Note

All claims expressed in this article are solely those of the authors and do not necessarily represent those of their affiliated organizations, or those of the publisher, the editors and the reviewers. Any product that may be evaluated in this article, or claim that may be made by its manufacturer, is not guaranteed or endorsed by the publisher.

## References

[B1] Babab E. (2007). Teachers' nonverbal behaviors and its effects on students. in The Scholarship of Teaching and Learning in Higher Education: An Evidence-Based Perspective, eds R. Perry and J. C. Smart (New York, NY: Springer), 201–61.

[B2] Baloch K. Akram M. W. (2018). Effect of teacher role, teacher enthusiasm and entrepreneur motivation on startup, mediating role technology. Arab. J. Bus. Manag. Rev. 7, 1–14.

[B3] Burić I. Moe A. (2020). What makes teachers enthusiastic: the interplay of positive affect, self-efficacy and job satisfaction. Teach. Teacher Educ. 89, 1–10. 10.1016/j.tate.2019.103008

[B4] Çevik Kiliç D. B. (2018). The relationship between the burnout levels of music teachers and their personalities. Int. Educ. Stud. 11, 38–54. 10.5539/ies.v11n2p38

[B5] Cui G. Yao M. Zhang X. (2017). The dampening effects of perceived teacher enthusiasm on class-related boredom: the mediating role of perceived autonomy support and task value. Front. Psychol. 8, 1–11. 10.3389/fpsyg.2017.00400 28367134 PMC5356221

[B6] Derakhshan A. (2021). The predictability of Turkman students' academic engagement through Persian language teachers' nonverbal immediacy and credibility. J. Teach. Persian Speak. Other Lang. 10, 3–26. 10.30479/jtpsol.2021.14654.1506

[B7] Dewaele J. M. Li C. (2020). Emotions in second language acquisition: a critical review and research agenda. For. Lang. World 1, 34–49.

[B8] Exeter D. J. Ameratunga S. Ratima M. Morton S. Dickson M. Hsu D.. (2010). Student engagement in very large classes: the teachers' perspective. Stud. High. Educ. 35, 761–775. 10.1080/03075070903545058

[B9] Frenzel A. C. Goetz T. Lüdtke O. Pekrun R. Sutton R. E. (2009). Emotional transmission in the classroom: exploring the relationship between teacher and student enjoyment. J. Educ. Psychol. 101, 705–716. 10.1037/a0014695

[B10] Hiver P. Al-Hoorie A. H. Mercer S. (2021). Student Engagement in the Language Classroom. Bristol: Multilingual Matters. 10.21832/9781788923613

[B11] Hsu L. (2010). The impact of perceived teachers' nonverbal immediacy on students' motivation for learning English. Asian EFL J. 12, 188–204.

[B12] Keller J. L. (2011). Advancing student success with competency points: elevating engagement and motivation in community college English composition students. Commun. Coll. J. Res. Pract. 35, 484–504. 10.1080/10668926.2010.515513

[B13] Keller M. M. Becker E. S. Frenzel A. C. Taxer J. L. (2018). When teacher enthusiasm is authentic or inauthentic: lesson profiles of teacher enthusiasm and relations to students' emotions. Aera Open 4, 1–16. 10.1177/233285841878296722956895

[B14] Keller M. M. Goetz T. Becker E. S. Morger V. Hensley L. (2014). Feeling and showing: a new conceptualization of dispositional teacher enthusiasm and its relation to students' interest. Learn. Instr. 33, 29–38. 10.1016/j.learninstruc.2014.03.001

[B15] Kunter M. Holzberger D. (2014). Loving teaching: research on teachers' intrinsic orientations, in Teacher Motivation: Theory and Practice, eds P. W. Richardson, S. Karabenick, and H. M. G. Watt (New York, NY: Routledge Taylor/ Francis), 83–99.

[B16] Lam S.-f. Jimerson S. Kikas E. Cefai C. Veiga F. H. Nelson B.. (2012). Do girls and boys perceive themselves as equally engaged in school? The results of an international study from 12 countries. J. School Psychol. 50, 77–94. 10.1016/j.jsp.2011.07.004 22386079

[B17] Lazarides R. Gaspard H. Dicke A.-L. (2019). Dynamics of classroom motivation: teacher enthusiasm and the development of math interest and teacher support. Learn. Instr. 60, 126–137. 10.1016/j.learninstruc.2018.01.012

[B18] Li C. (2020). A Positive Psychology perspective on Chinese EFL students' trait emotional intelligence, foreign language enjoyment and EFL learning achievement. J. Multilingual Multicultural Dev. 41, 246–263. 10.1080/01434632.2019.1614187

[B19] MacIntyre P. D. Gregersen T. Mercer S. (2019). Setting an agenda for positive psychology in SLA: theory, practice, and research. Modern Lang. J. 103, 262–274. 10.1111/modl.12544

[B20] Mercer S. (2019). Language learner engagement: setting the scene, in Second Handbook of English Language Teaching, ed X. Gao (Cham: Springer), 643–660.

[B21] Neel C. G.-O. Fuligni A. (2013). A longitudinal study of school belonging and academic motivation across high school. Child Dev. 84, 678–692. 10.1111/j.1467-8624.2012.01862.x 23002809

[B22] Oga-Baldwin W. L. Q. (2019). Acting, thinking, feeling, making, collaborating: the engagement process in foreign language learning. System 86:102128. 10.1016/j.system.2019.102128

[B23] Patrick B. C. Hisley J. Kempler T. (2000). What's everybody so excited about?: the effects of teacher enthusiasm on student intrinsic motivation and vitality. J. Exp. Educ. 68, 217–236. 10.1080/00220970009600093

[B24] Pishghadam R. Derakhshan A. Zhaleh K. Habeb Al-Obaydi L. (2021). Students' willingness to attend EFL classes with respect to teachers' credibility, stroke, and success: A cross-cultural study of Iranian and Iraqi students' perceptions. Curr. Psychol. 40, 1–15. 10.1007/s12144-021-01738-z

[B25] Pogue L. L. AhYun K. (2006). The effect of teacher nonverbal immediacy and credibility on student motivation and affective learning. Commun. Educ. 55, 331–344. 10.1080/03634520600748623

[B26] Shaw J. Kominko S. Terrion J. (2015). Using lecture tools to enhance student–instructor relations and student engagement in the large class. Res. Learn. Technol. 23, 1–13. 10.3402/rlt.v23.27197

[B27] Shoshani A. Eldor L. (2016). The informal learning of teachers: learning climate, job satisfaction and teachers' and students' motivation and well-being. Int. J. Educ. Res. 79, 52–63. 10.1016/j.ijer.2016.06.007

[B28] Skinner E. A. Pitzer J. R. (2012). Developmental dynamics of student engagement, coping and everyday resilience, in Handbook of Research on Student Engagement, eds S. L. Christenson, A. L. Reschly and C. Wylie (New York, NY: Springer), 21–44.

[B29] Ulmanen S. Soini T. Pietarinen J. Pyhältö K. (2016). Students' experiences of the development of emotional engagement. Int. J. Educ. Res. 79, 86–96. 10.1016/j.ijer.2016.06.003

[B30] Umbach P. D. Wawrzynski M. R. (2005). Faculty do matter: the role of college faculty in student learning and engagement. Res. High. Educ. 46, 153–184. 10.1007/s11162-004-1598-1

[B31] Wang P. (2017). Looking beyond the ELT approach in China's higher education from the perspective of bilingual education: immersion, content-based instruction or something else? Int. J. Bilingual Educ. Bilingualism 20, 102–114. 10.1080/13670050.2015.1037239

[B32] Wang Y. L. Derakhshan A. (2021). A book review on investigating dynamic relationship among individual difference variables in learning English as a foreign language in a virtual world [J]. System. 10.1016/j.system.2021.102531

[B33] Witkowski P. Cornell T. (2015). An investigation into student engagement in higher education classrooms. J. Scholar. Teach. 10, 56–67. 10.46504/10201505wi

[B34] Witt P. L. Wheeless L. R. (2001). An experimental study of teachers' verbal and nonverbal immediacy and students' affective and cognitive learning. Commun. Educ. 50, 327–342. 10.1080/03634520109379259

[B35] Xie F. Derakhshan A. (2021). A conceptual review of positive teacher interpersonal communication behaviors in the instructional context. Front. Psychol. 2021:708490. 10.3389/fpsyg.2021.708490 34335424 PMC8319622

[B36] Yin H. Wang W. (2016). Undergraduate students' motivation and engagement in China: an exploratory study. Assess. Eval. High. Educ. 41, 601–621. 10.1080/02602938.2015.1037240

[B37] York D. (2013). Investigating a Relationship Between Nonverbal Communication and Student Learning. (Doctoral Dissertation), Lindenwood University, Saint Charles, MO, United States.

[B38] Zhang Q. (2013). Assessing the effects of instructor enthusiasm on classroom engagement, learning goal orientation, and academic self-efficacy. Commun. Teacher 28, 44–56. 10.1080/17404622.2013.839047

[B39] Zhang Z. Hu W. McNamara O. (2015). Undergraduate student engagement at a Chinese university: abase study. Educ. Assess. Eval. Accountabil. 27, 105–127. 10.1007/s11092-015-9213-x

[B40] Zhu H. Arnold K. (2013). Understanding student engagement and achievement in Chinese universities: a study of Beijing college students. Econ. Educ. Res. 11, 1–16. 10.1163/22125868-12340014

